# Variation in the chemical composition of wheat straw: the role of tissue ratio and composition

**DOI:** 10.1186/s13068-014-0121-y

**Published:** 2014-08-20

**Authors:** Samuel RA Collins, Nikolaus Wellner, Isabel Martinez Bordonado, Andrea L Harper, Charlotte N Miller, Ian Bancroft, Keith W Waldron

**Affiliations:** Institute of Food Research, Norwich Research Park, Colney, Norwich, NR4 7UA UK; John Innes Centre, Norwich Research Park, Colney, Norwich, NR4 7UH UK; Present address: Department of Biology, University of York, Wentworth Way, Heslington, York, YO10 5DD UK

**Keywords:** Wheat straw, Lignocellulose, Composition, Biomass, Ethanol yields

## Abstract

**Background:**

Wheat straw is an attractive substrate for second generation ethanol production because it will complement and augment wheat production rather than competing with food production. However, like other sources of lignocellulosic biomass, even from a single species, it is heterogeneous in nature due to the different tissues and cell types, and this has implications for saccharification efficiency. The aim of this study has been to use Fourier transform infrared (FTIR) spectroscopy and Partial least squares (PLS) modelling to rapidly screen wheat cultivars for the levels of component tissues, the carbohydrate composition and lignin content, and the levels of simple cross-linking phenolics such as ferulic and diferulic acids.

**Results:**

FTIR spectroscopy and PLS modelling was used to analyze the tissue and chemical composition of wheat straw biomass. Predictive models were developed to evaluate the variability in the concentrations of the cell wall sugars, cell wall phenolics and acid-insoluble lignin. Models for the main sugars, phenolics and lignin were validated and then used to evaluate the variation in total biomass composition across 90 cultivars of wheat grown over two seasons.

**Conclusions:**

Whilst carbohydrate and lignin components varied across the varieties, this mainly reflected differences in the ratios of the component tissues rather than differences in the composition of those tissues. Further analysis indicated that on a mol% basis, relative levels of sugars within the tissues varied to only a small degree. There were no clear associations between simple phenolics and tissues. The results provide a basis for improving biomass quality for biofuels production through selection of cultivars with appropriate tissue ratios.

**Electronic supplementary material:**

The online version of this article (doi:10.1186/s13068-014-0121-y) contains supplementary material, which is available to authorized users.

## Background

Lignocellulosic biomass is recognized as an important resource for the production of renewable energy, biofuels and biochemicals [1]. Lignocellulosic biomass may be obtained from many sources, from waste streams in forestry and agriculture through to energy crops grown for the purpose. However, there is concern that cultivation of the latter may result in competition with food production [2]. Wheat straw is produced globally in large quantities [3]. It is an attractive substrate for second generation ethanol production because it will complement and augment wheat production rather than competing with food production. As a result there has been much research to develop biorefining technologies to pretreat, enzymatically saccharify and ferment the constituent sugars of wheat straw to produce ethanol and other products. Lignocellulosic biomass, even from a single species such as wheat, is heterogeneous in nature [4,5]. The chemical compositions may vary according to constituent agronomic conditions, location and local climate [6], in addition to heritable variation. This will have an impact on the saccharification potential for production of ethanol [7]. Assessing the chemical composition of lignocellulosic biomass is therefore necessary for the optimization of biorefining approaches [6]. Compositional analysis is also needed to provide a basis for future breeding improvements not only for biofuel production, but also for other potentially renewable products that can be produced from straw components. These include fibres [3], functional hemicelluloses [8] and phenolics such as ferulic acid [9]. Whilst many cultivars of wheat have been developed in order to optimize grain quality and yield for human and animal consumption, there has been little emphasis on developing the non-food components for biorefining purposes. Unfortunately, wet chemical analysis of large numbers of different samples is expensive and time consuming. Hence there have been several studies to evaluate the potential utilization of spectroscopy in measuring (rapidly) the composition of feedstock. For example Liu *et al*. [6] investigated the use of Fourier transform near infrared spectroscopy (FT-NIR) techniques to evaluate variability in biomass chemical composition in corn stover and switch grass. Lindedam *et al*. [10] demonstrated that FT-NIR spectra could be used to screen sugar release and chemical composition in 20 cultivars of wheat straw and further demonstrated considerable varietal differences in sugar yield [11]. Lomborg *et al*. [12] used 44 samples of wheat straw to demonstrate the use of near infrared spectroscopy in quantifying key carbohydrate components and lignin. Tamaki and Mazza [13,14] demonstrated the potential to use Fourier transform (mid) infrared (FTIR) spectroscopy to develop partial least squares (PLS) models for predicting carbohydrates, ash and extractives in two cultivars of wheat and triticale, and used a similar technique to measure lignin in wheat straw. In spite of these models, only one [10] has been used to actually screen a range of wheat cultivars, and in that case a very large degree of variation was found in the results which related to digestible sugars rather than original composition.

The aim of this study has been to use FTIR and PLS modelling to develop a rapid method of evaluating the levels of component tissues, the carbohydrate composition and lignin content, and the levels of simple cross-linking phenolics such as ferulic and diferulic acids. This approach has been used to screen biomass samples from 90 cultivars of wheat grown at several locations over two seasons, and assess the variation within the lignocellulose, as well as the correlations between components measured.

## Results and discussion

### Composition of wheat plant tissues

**Figure 1:**
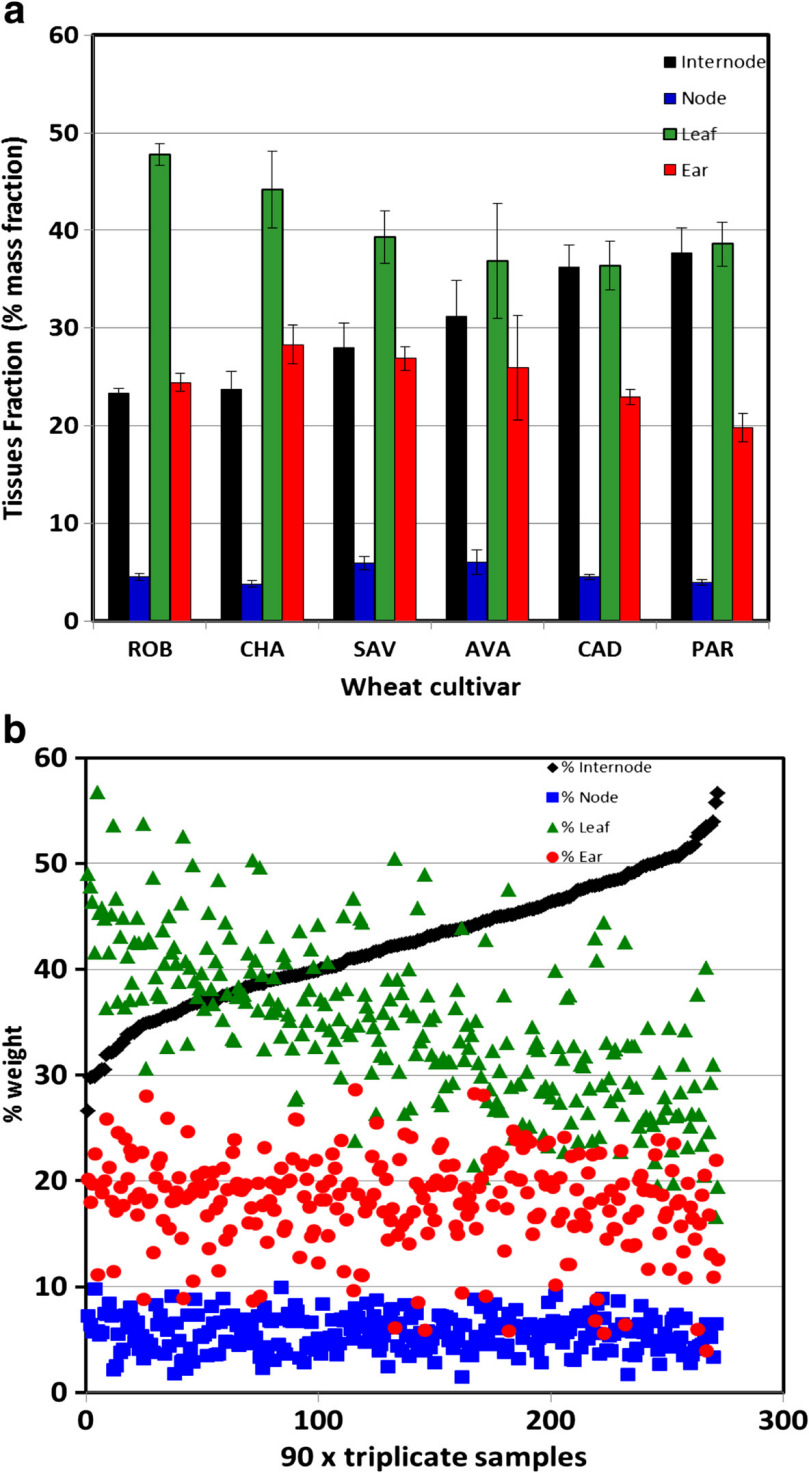
**Distribution of tissue types (by weight) within wheat samples: (a) means with standard errors for the six cultivars Cadenza (CAD), Paragon (PAR), Savanah (SAV), Robigus (ROB), Charger (CHA) and Avalon (AVA); (b) PLS-model-derived data for 2011 field-grown wheat samples.**

Wheat straw biomass consists mainly of lignocellulosic materials, but the different parts of the plant have quite distinct variations in their compositions [4,5,15,16]. In this study, six selected cultivars, Cadenza (CAD), Paragon (PAR), Savanah (SAV), Robigus (ROB), Charger (CHA) and Avalon (AVA) were evaluated for their tissue yields and compositions. The accessions used for model development were selected from available seed stocks according to their morphology and growth habit. They represent both winter and spring wheat types, and their morphology includes solid and hollow straw types and a range of plant heights. It was anticipated that these accessions would capture diversity for both tissue composition and cell wall chemistry traits to facilitate the development of FTIR models for screening these parameters. The proportions of the air-dried component tissues are shown in Figure 1a. The quantity of node tissue was small and showed little variation between the plants, whilst the internode and leaf tissues comprised the bulk of the biomass and varied considerably. In ROB, the leaf tissue comprised about 50% of the plant materials and was twice that of the internode tissue at 25%. In contrast, in PAR and CAD the levels of internode and leaf tissues were similar at about 40%. The ear tissue was also significant at between 20 and 28% (% mass fraction), but showed no trend in relation to other tissues. For comparison, Jacobs *et al*. [5] in evaluating the mass balance of tissues in winter wheat (Madsen cultivar) found the ratios of internode:node:leaf to be 53.2:9.1:37.3% respectively, and Pearce *et al*. [15] reported 50:8:42% respectively (on an unnamed cultivar).

The dried tissues were milled (<250 μm); moisture contents were measured and found to be constant at 7-8% (w/w). The milled materials were chemically analyzed in this state without any treatments to remove any extractable substances (and were thus representative of raw whole material). The amounts of rhamnose, fucose, arabinose, xylose, mannose, galactose, glucose, uronic acid (as anhydro sugar equivalents) and a range of functionally important cell wall phenolics including ferulic acid, a range of diferulic acids, coumaric acid and lignin are presented in Additional files 1 and 2: Tables S1 and S2.

The analyses showed the expected distinct differences in tissue composition. Total carbohydrate levels were highest in ear tissues at 62% w/w (67% w/w dry matter (DM)) in SAV and lowest in leaf tissues at 46% w/w (49% DM) in CHA. The main sugars were glucose and xylose. Glucose was the dominant cell wall sugar and its content was highest in the internode, ranging from 29% w/w (31% w/w DM) in AVA to 44 w/w (48% w/w DM) in CAD, and lowest in leaf tissue, consistent with earlier studies [4] but demonstrating significant variation between plants. The xylose content of internode, leaf and node tissues was generally half the level of glucose, but was significantly (approximately 30%) higher in the ear than in the other tissues. Arabinose at between 1 and 3% DM was lowest in the internode (where the xylans are poorly branched) and about double that level in all other tissues, reflecting the presence of highly substituted arabinoxylans. Lignin (corrected for ash) content was highest in the internode, and lowest in the node, probably reflecting the requirement for the node to undergo controlled extension to address lodging disturbances.

Uronic acid was present in all tissues, and will have been derived predominantly from glucuronic acid found in glucuronoarabinoxylans [8]. However some will have originated from galacturonic acid in the small quantities of pectic polysaccharides found particularly in the leaf tissues. This was clearly indicated by the small but measurable levels of rhamnose, which was highest in leaf tissues and lowest in internode and ear tissues. The internode-derived uronic acid was generally between 4 and 5% dry mass fraction. However, the leaf uronic acid component varied considerably in the leaves and nodes of the modelled tissues, ranging between 6 and 10%. Mannose was present at its highest levels in the node and internode compared with leaf tissue, and lower still in ear tissue. These values reflect the predominance of lignocellulose or hemicellulose in the stem. It is possible that some mannose may have been derived from hydrolysis and reduction of any residual sucrose present in the tissues.

Lignin was measured gravimetrically and corrected for ash in all tissues (Additional files 1 and 3: Table S1 and S3). There was considerable variation in content, which related to both tissue type and cultivar. In nearly all cultivars lignin was at its highest level in the internode tissue, but varied from over 20% w/w in ROB down to under 14% w/w in AVA. The level of lignin in the other tissues was generally within 20% of that of the internode value, but their relative levels also varied between cultivars.

For comparison, Additional file 3: Table S3 shows published values for cell wall sugars and lignin from whole wheat straw and constituent tissues from a number of studies over the last 25 years. Notwithstanding minor variations in the methodologies, it is important to note that on a dry matter basis, the level of glucose in whole straw ranges from under 30% w/w DM [5] to over 40% w/w DM [10], and is reported as high as 44.8% w/w DM in internode tissues [4], although that calculation was gravimetric and by difference. Xylose and lignin values vary considerably also (18 to 24% and 14 to 25% w/w DM respectively). Such variation is consistent with that found in the compositions of component tissues of the six cvs reported in this study.

Phenolic esters were analyzed across the four tissues in the six plant varieties for modelling. The distributions are shown in Additional file 2: Table S2. The main phenolic ester was p-coumaric acid (pCA) which, in all the cultivars, was highest in the node tissues, ranging from 0.5% w/w in PAR up to 1.8% w/w in ROB. In most cultivars, the leaf tissue exhibited the lowest level of pCA, ranging from 0.25% w/w to 0.5% w/w in ROB, CHA and AVA. The distribution of pCA in the internode and ear tissues varied widely. The next most prominent phenolic ester was trans-ferulic acid (FA). The levels differed considerably between the cultivars, but were distributed in a similar manner between the tissues. FA ranged from under 0.3% w/w in PAR to up to 0.6% in CHA and AVA. The other main phenolic moieties comprised diferulic acid species of which the 8-0-4’DiFA was generally highest in leaf and ear tissues at about 0.1% (w/w) and lowest in internode tissues. Small but significant levels of other phenolics were identified, including vanillic acid and vanillin. For each cultivar, the standard errors for the phenolics data were quite noticeable (Additional file 2: Table S2). However, this was not due to experimental error, but due to strong variability between different replicate plants used in building the model. For individual plants, the errors were small (in the region of 2 to 4% of the means). This contrasts with the sugars data which gave low variation between plants of a specific cultivar (Additional file 1: Table S1).

### Development of partial least squares tissue models from Fourier transform infrared spectra

**Figure 2:**
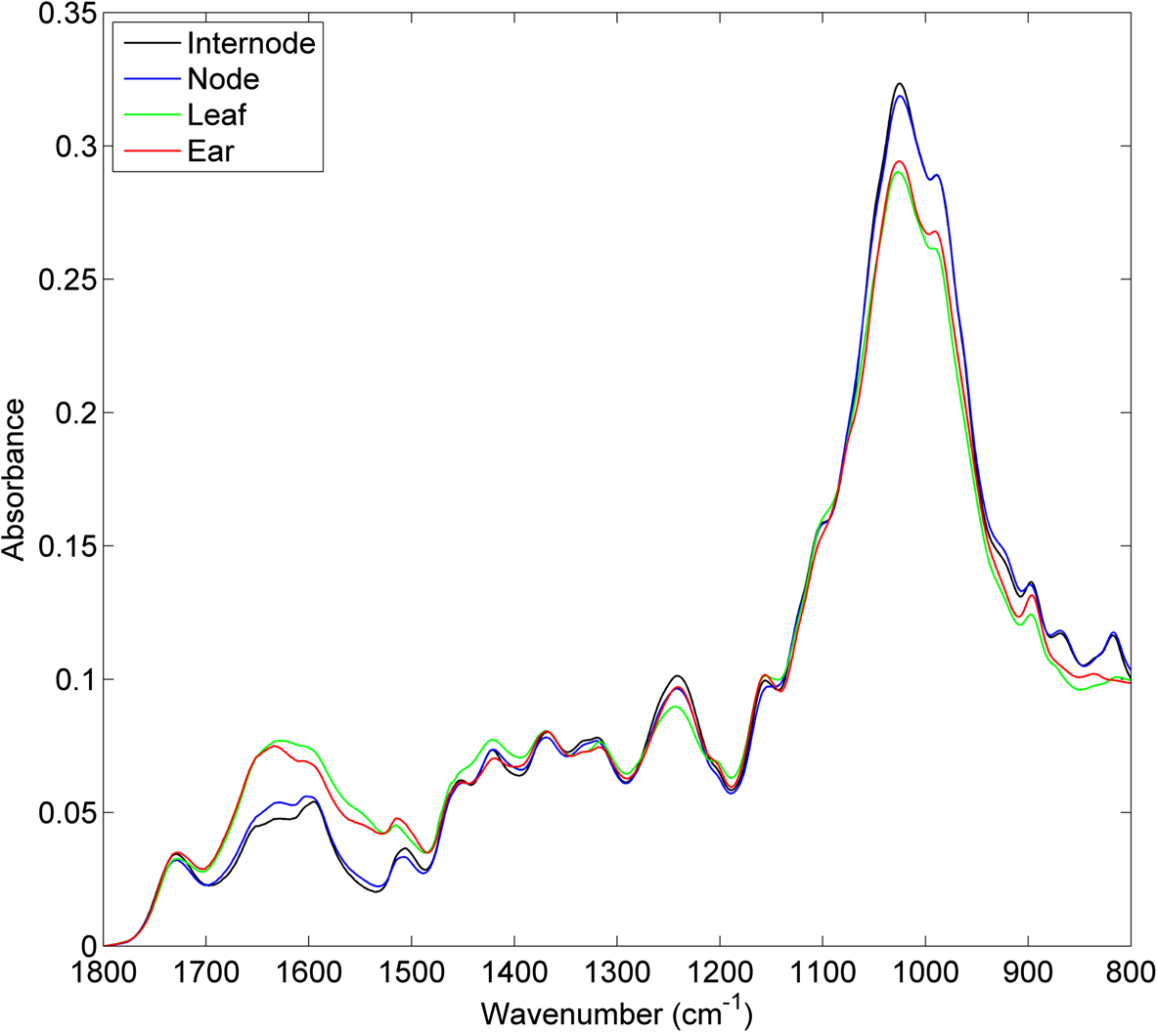
**FTIR-ATR spectra of the four different tissues of wheat straw (cv Avalon) (Averaged spectra from four plants, with five area-normalized replicate spectra each).**

Figure 2 shows representative Fourier transform infrared attenuated total reflectance (FTIR-ATR) spectra of the separated tissue types from wheat straw internode and node, leaf, and ear spikelets. The varying chemical composition of the tissues was reflected in distinct variations between their FTIR spectra. The spectra of nodes and internodes showed more prominent bands at 1590 and 1510 cm^−1^ than those of the leaf and ear spikelets. These bands are generally attributed to lignin-like moieties, although this did not directly reflect differences in Klason ash-corrected lignin. In contrast, broad absorption bands at 1630 and 1550 cm^−1^ indicated a higher amount of protein in the latter two tissues, and the carbohydrate bands at 1020 and 990 cm^−1^ were relatively smaller. The spectra of these two tissues also lacked two smaller bands at 860 and 820 cm^−1^ in the anomeric region of carbohydrates. These spectral differences were consistent across lines, although band intensities varied between different samples.

Since whole wheat straw biomass is a mixture of these tissues, it would be reasonable to assume that the spectrum of the whole wheat is a linear combination of the component spectra (the models were derived from tissues dissected from whole plants which had not been subjected to harvesting-related losses of loose and friable parts like leaves). Hence a PLS model was created to quantify the relative amounts of these tissues in wheat straw biomass. The assumption was then justified by confirming that the spectrum of a measured mixture of the four tissue powders was equivalent to one obtained by digitally adding the four %-weighted spectra of the individual components (results not shown). Models made from the raw spectra performed reasonably well. However some of the hemicellulose sugars, notably xylose, exhibited a constant underestimation bias in test set predictions. This was successfully addressed by using first derivative spectra to eliminate nonlinear baseline effects. In contrast, a fourth order polynomial spline baseline correction did not improve the predictions.

**Figure 3:**
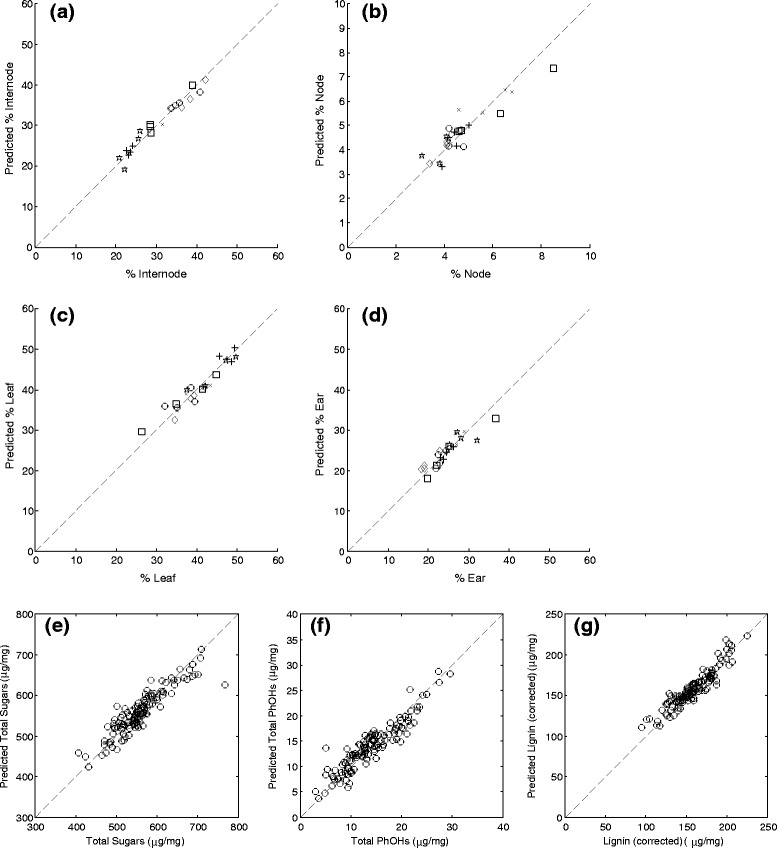
**PLS model-predicted data versus actual data: (a-d) PLS predicted versus actual weight percentage of individual tissue types in whole plants from six different wheat cultivars (square: Avalon, circle: Cadenza, Star: Charger, diamond: Paragon, plus: Robigus, cross: Savannah; average of four plants for each cv).** PLS predicted versus actual contents plotted for **e)** total sugars, **f)** total phenolics and **g)** corrected lignin in the calibration set (samples listed in Table 1, five technical replicate predictions for each sample averaged).

Examples of correlations between measured and predicted values for tissue proportions are shown in Figure 3a-d. Satisfactory predictions with relative errors between 6 and 8% could be made for the relative amounts of internode, ear and leaf tissue. The prediction error for the amount of node was adequate but greater (12%), firstly, because this was by far the smallest constituent and secondly, because the similarity of its chemical composition with the internode is likely to have caused some material to be misallocated.

### Development of partial least squares chemical models from Fourier transform infrared spectra

A total of 28 chemical constituents of the wheat straw were modelled using the calibration sample set listed in Table 1. Figure 3e-g shows the calibration curves for total sugars, phenolics and lignin as examples. Table 2 provides an overview of the input data, number of factors used and the error for all PLS models.

**Table 1:** **Calibration samples used for the models**

	**CV**	**Whole plants**	**Tissues**	**Replicates**	**Spectra**
Field	Consort	1	-	5	5
	Deben	1	-	5	5
	Etoile-de-Choisy	1	-	5	5
	Extrem	1	-	5	5
	Hustler	1	-	5	5
	Orlando	1	-	5	5
	Sperber	1	-	5	5
	Steadfast	1	-	5	5
Greenhouse					
	Avalon	4*	4	5	100
	Cadenza	4*	4	5	100
	Charger	4*	4	5	100
	Paragon	4*	4	5	100
	Robigus	4*	4	5	100
	Savannah	4*	4	5	100

*whole sample spectra calculated from the tissues.

**Table 2:** **Calibration range and prediction errors of the partial least squares models RMSEC: root mean square error of prediction**

**Sugars**	**Minimum ** **(μg/mg)**	**Maximum ** **(μg/mg)**	**Average ** **(μg/mg)**	**SD**	**RMSEC**	**Factors**	**RMSEC as % of average**
Total sugars	425.36	628.92	537.29	35.84	21.43	10	4
Rhamnose	0.31	2.1	1	0.41	0.22	5	22
Fucose	−0.23	1.42	0.49	0.25	0.26	12	53
Arabinose	6.47	46.06	25.57	8.02	2.19	5	9
Xylose	103.24	224.83	161.4	27.61	9.14	5	6
Mannose	0.87	31.64	9.86	7.24	3.41	5	35
Galactose	2.62	11.53	7.22	2.75	0.94	4	13
Glucose	228.49	359.41	300.5	30.91	17.43	10	6
Uronic acids	19.97	48.61	31.26	6.5	3.9	4	12
**Phenolics**	**Minimum ** **(μg/mg)**	**Maximum ** **(μg/mg)**	**Average ** **(μg/mg)**	**SD**	**RMSEC**	**Factors**	**RMSEC as % of average**
Klason lignin	107.63	241.21	168.91	21.79	14.4	10	9
Acid insoluble ash	1.47	28.89	14.3	6.27	6.09	10	43
Corrected lignin	101.44	232.16	154.79	21.31	11.63	10	8
Protocatechuic aldehyde	0.002	0.013	0.007	0.002	0.002	10	35
p-OH-Benzoic acid	−0.011	0.364	0.105	0.064	0.061	10	58
Vanillic acid	0.079	0.389	0.205	0.061	0.051	10	25
p-OH-Benzaldehyde	0.002	0.301	0.102	0.056	0.032	10	32
Truxillic acid (CA)	−0.004	0.021	0.01	0.005	0.005	10	46
Truxillic acid (FA)	0.007	0.032	0.02	0.004	0.005	10	25
Vanillin	−0.011	0.423	0.216	0.082	0.069	10	32
t-p-Coumaric acid	1.445	20.043	7.501	3.565	2.098	10	28
8,8'-DiFA (aryl tetralin form)	0	0	0	0	0		
t-Ferulic acid	1.669	7.942	4.462	1.059	1.015	10	23
8,8'-DiFA	0.005	0.149	0.078	0.026	0.019	10	25
8,5'-DiFA	−0.035	1.004	0.459	0.23	0.117	10	26
5,5'-DiFA	0.047	0.389	0.202	0.08	0.035	10	17
8-O-4'-DiFA	0.172	1.066	0.655	0.21	0.103	10	16
8,5'-DiFA (benzofuran form)	0.091	0.279	0.164	0.034	0.033	10	20
Total phenolic acids	5.637	32.61	14.356	4.558	3.345	10	23
Total phenolic acids (plus estimated unknown peaks)	5.25	32.659	14.438	4.591	3.344	10	23
**Tissues**	**Minimum**	**Maximum**	**Average**	**SD**	**RMSEC**	**Factors**	**RMSEC as % of average**
Internode	18.00%	42.70%	30.00%	6.10%	1.70%	6	6
Node	3.00%	7.70%	4.80%	1.00%	0.60%	9	12
Leaf	27.90%	52.80%	40.50%	5.40%	2.20%	6	5
Ear	17.30%	33.70%	24.70%	3.60%	1.90%	6	8

The ‘total sugars’ content was found to model reasonably well with a Root mean square error of calibration (RMSEC) value of 21.43 mg/g, representing a 4% relative error on the average total sugars content. Glucose was predicted with a RMSEC of 17.43 mg/g, representing a 6% relative error on the average glucose content. The models for hemicellulosic and pectin components showed some variability. Whereas arabinose and xylose could be modelled similarly to glucose, with calibration errors of 9% and 6% respectively, rhamnose and galactose models had larger prediction errors (13 to 22%). Uronic acids, encompassing both galacturonic acids from pectin and glucuronic acid from hemicelluloses, were modelled with a relative prediction error of 12%. The worst performing carbohydrate models were for the minor sugars such as mannose, with a relative prediction error of 35%, and fucose with an error of 53%. The error for the latter component meant that the fucose PLS model could not give a usable prediction. Lignin models gave good predictions, with a 9% relative error, which improved slightly to 8% after correction for acid-insoluble ash.

Para-coumaric acid (pCA) and FA made up the bulk of the phenolic compounds. Most of the other components were present only in very small amounts (much less than 1% of total dry matter). Their spectral signatures are relatively similar and could also be obscured by the much bigger lignin bands in the region of 1400 to 1640 cm^−1^. Therefore it was quite surprising that PLS models could be made to work for a large number of these (Table 2), albeit with relatively high prediction errors in the order of 20 to 30%. Such an error is not surprising in view of the level of variability between individual plants discussed above.

The quality of modelling can be compared with that of other recent, relevant studies. Lomborg *et al*. [12] used a wide range of approximately 100 whole straw samples (down-sampled, milled to 1 mm) from a variety of sources and different seasons to explore the use of FT-NIR spectroscopy in determining chemical composition. They reported %RMSEP (root mean square of prediction) values of 11% for glucan and xylan, 13% for arabinan and 12% for lignin, using 5, 5, 4 and 7 PLS factors respectively. This relied on heavy use of outlier rejection (as much as 18% for lignin). A subsequent FT-NIR rapid analysis study by Liu *et al*. [6] on corn and switchgrass (not wheat straw) gave lower relative errors of 1.99, 2.3, 10.96, 7.53, 6.65, 3.62 and 13.95% for glucan, xylan, galactan, arabinan, mannan, lignin and ash. FTIR has an advantage over FT-NIR in that much more chemical information is shown by the fundamental vibrations.

Using FTIR spectroscopy, Tamaki and Mazza [13] gave relative prediction errors of 1.11% and 1.35% for total glycans and glycan, 1.8% for xylan, 9.15% for galactan, 6.95% for arabinan and 23.8% for mannan. Tamaki and Mazza [14] also reported relative prediction errors of 2% for lignin (10 to 11 PLS factors for glucan and xylan, 6 to 7 for arabinan and galactan, and 9 to 12 for lignin). However, although their study involved whole straw samples for triticale and wheat collected over two seasons at different locations in Canada, they used only two to three cultivars each, demonstrating inherently much less variation. They explained the lower accuracy for smaller components by the low concentrations and relatively greater errors in their chemical analysis.

In the present study our results have shown similar trends, with the minor components like mannose giving worse predictions than the predominant glucose and lignin. The prediction errors in our models are similar to those of Liu *et al*. [6] and Lomborg *et al*. [12], and a little higher than in the Tamaki studies [13,14]. However, compared to Tamaki and Mazza [13,14], this study used fewer PLS factors. Increasing the number of the PLS factors would have improved the RMSEC values obtained by internal cross-validation. However, overfitting the calibration set would have made the prediction errors for independent samples worse. Averaging the input spectra did not increase the prediction accuracy because the replicate infrared spectra were already closely grouped, and very few spectra could be considered as outliers. In addition, we observed a marked increase in accuracy when we reduced the number of different wheat lines in the calibration set, and individual lines tended to model extremely accurately. Nevertheless, the aim of this study was to evaluate variation across a wide range of samples. Hence the models were developed with six wheat lines that had been preselected for high phenotypic variability in order to maximize the potential for downstream evaluation of field-grown cultivars (below).

### Assessment of variation in 90 varieties of field-grown wheat

A set of 90 field-grown wheat cultivars with a spread of genetic variation was grown over two seasons (see Materials and Methods). The whole plants were carefully harvested (to avoid loss of friable tissues such as dry crumbly leaves) and milled to less than 250 μm particle size. Moisture content was between 7 and 8% (w/w). The milled samples were analyzed by FTIR and the spectra were fed into the PLS models. The results were then used to assess variation of chemical parameters across the cultivars, and correlations between chemical and physical parameters. In parallel, an additional set of plants (five replicates) were assessed for key physical parameters (dimensions and mechanical properties).

### Correlations between different chemical moieties in the wheat lines

**Figure 4:**
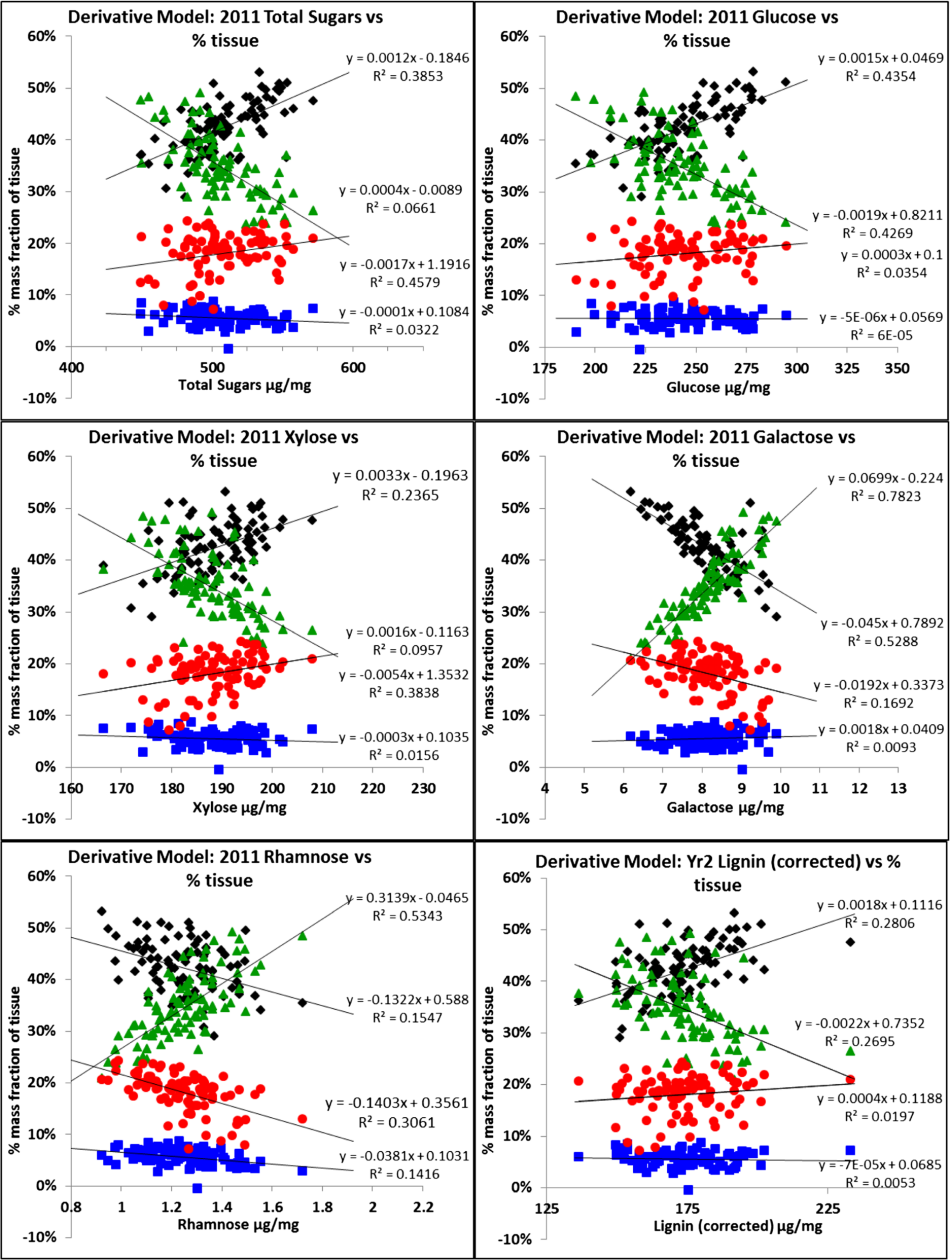
**Correlations of component concentrations with tissue type.**

A correlation table was created to assess the interrelationships between chemical data calculated from the models, and selected physical data assessed post-harvest (Additional file 4: Table S4). The correlation table highlighted areas of positive (green) and negative (red) correlation. In this paper, the data discussed are those of 2011. However, the trends described were also shown in the 2010 results. Height and stem length were, unsurprisingly, closely correlated (0.993). Both of these characteristics were positively correlated with the quantities of internode tissues (0.753 and 0.754) but negatively with the proportion of leaf tissues (−0.630 and −0.629 respectively). There was no significant association with the quantity of ear and node tissue. Figure 1b shows the percentage tissue weights of the 270 replicate samples from 2011 which have been ordered in increasing weight of internode tissue. The level of internode tissue ranges from under 30% air-dry weight to nearly 60% and the proportion of leaf is inversely related. Node and ear tissues show no obvious trends relative to the other tissues. Additional file 4: Table S4 also includes correlations between these physical parameters and the chemical compositional data derived from the chemical model. Of particular note were the relationships between the tissue types (which had been independently derived from the tissue model) and several key chemical components. The most prominent of these are presented as correlation plots in Figure 4. The levels of total sugars, glucose and xylose, and to a lesser extent lignin, are positively correlated with the proportions of stem tissue, and negatively correlated with the proportions of leaf tissue. There is little correlation with ear or node tissues. In contrast, the levels of galactose and rhamnose are negatively correlated with stem tissue and positively correlated with the proportion of leaf tissue, reflecting the more pectin-rich cell wall chemistry. Again, there is little correlation with node or ear. These results are consistent with the observed correlation between glucose, total sugars and xylose with stem height. They also strongly indicate that much of the variation in carbohydrate and lignin chemistry across the different cultivars is dependent on the proportions of the component tissues, particularly the ratios of internodes and leaves.

The interpretation of phenolic data was less clear, partly because of the higher levels of error in the model as discussed above. However, the correlation table suggested a positive correlation between 8-0-4’DiFA and leaf, but negative with internode tissue. These data are supported by the chemical analysis of the six cultivars used for developing the PLS models; the results (Additional file 2: Table S2) show that 8-0-4’DiFA is often highest in the leaf tissue. The conclusion is strengthened further by the observation that of all the phenolics, the 8-0-4’DiFA gave the lowest RMSEC as a percentage of the average at 16%. The interpretation is consistent with the observation that the 8-0-4’DiFA is inversely correlated with lignin, which is present in low levels in the leaf tissue but high in the internode where the 8-0-4’DiFA is low. Interestingly, in spite of the relatively high RMSEC values, many of the diferulic acids showed good correlations with each other, reflecting the commonality of synthesis during peroxidative cross-linking within the plant cell walls.

Several additional positive and negative correlations between the chemical components could also be detected. Lignin (corrected or not) was negatively correlated with nearly all of the diferulates (Additional file 4: Table S4, −0.3 to −0.77), presumably reflecting the lack of a phenolic cross-linking requirement in lignified stem tissues and the reduced levels of lignin in phenolic-cross-linked leaf tissues. However, lignin was highly correlated with xylose (0.73) but not arabinose, reflecting the higher degree of lignification in xylan-rich cell walls. Vanillin was highly correlated with ferulic acid, probably reflecting the flux through phenolic synthesis pathways common to both moieties [17].

**Figure 5:**
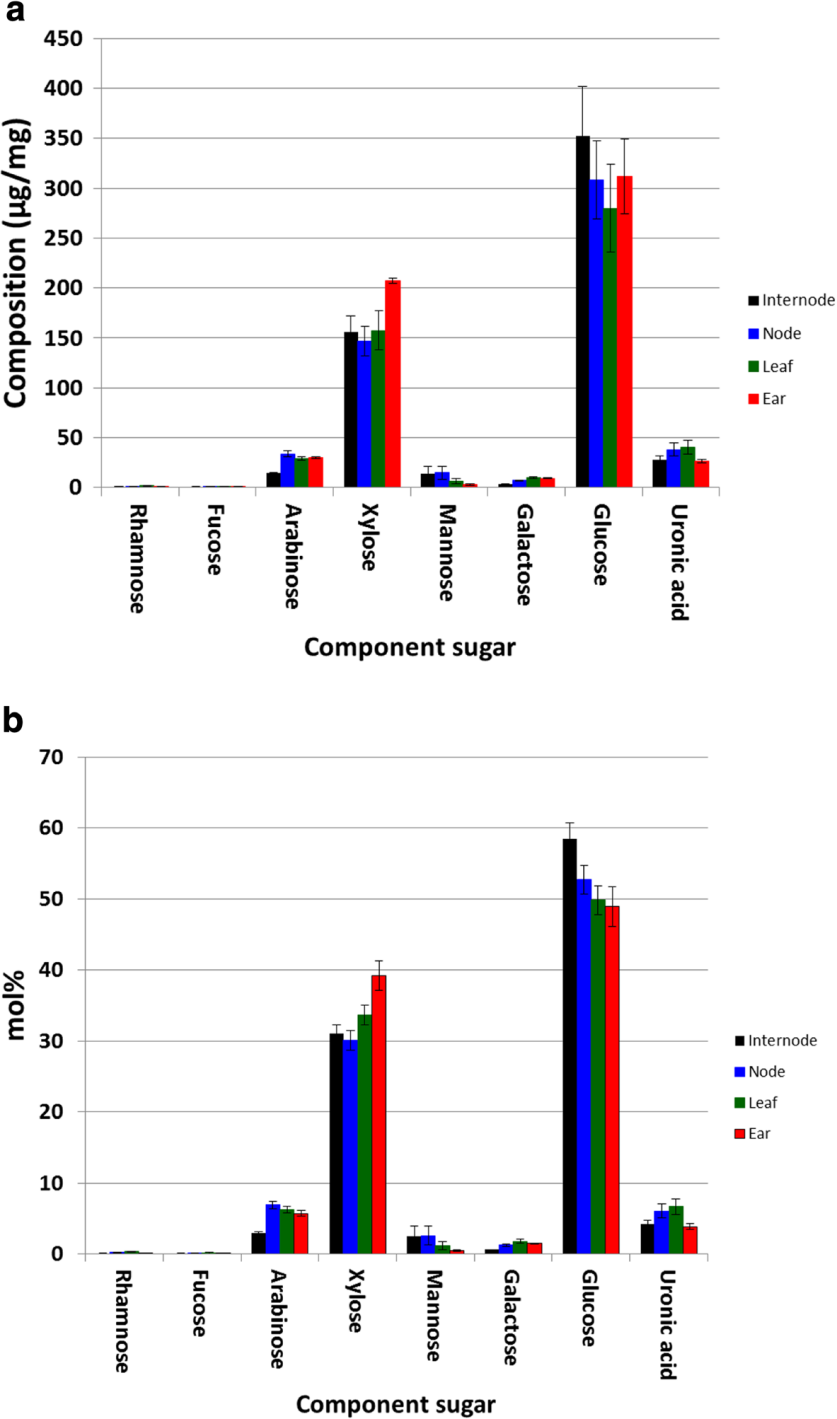
**Variation in cell wall sugar compositions in tissues of six wheat cultivars: (a) Variation in w/w composition (b) Variation in mol% composition.**

Whilst the results highlighted the important role of the tissue ratios in determining the overall straw chemical composition, the modelled data could not provide any information on variation within the tissues across the cultivars. This is because the PLS models, whilst enabling the levels of tissues and chemical components to be assessed in whole plant material, could not provide any indication of the chemical compositions of the individual tissues. However, such variation could be evaluated from the chemical analyses of the individual tissues from six lines used in developing the models. Figure 5a shows the mean values for carbohydrate compositions in the different tissues from the modelling lines. The error bars show significant variation, particularly for glucose, reflecting a spread in the composition (as indicated also in Table 2). However, presentation of the sugars data as mol% (Figure 5b) shows very little variation. Hence, although the overall levels of individual sugars in any one tissue vary between cultivars on a total weight basis, the ratios between the component sugars are almost unchanged. This suggests that the cell wall carbohydrate chemistry within wheat organs is highly conserved. The variation in the overall composition is thus attributable to changes in the relative levels of non-carbohydrate components such as lignin, ash, and extractives (not assessed) on an individual tissue basis, strongly modulated by the relative ratios of the tissues themselves. In addition, since significant quantities of leaves are often lost during harvest due to conversion to dust, it is likely that further variability will result. Variation in tissue and chemical compositions is likely to have a significant impact on the way in which the straw is best exploited, whether it be for bioethanol production or for the extraction of other components, such as hemicelluloses and phenolics. Zhang *et al*. [18] have demonstrated that pure leaf fractions of wheat straw were much less recalcitrant compared to pure stem, and were easily digested by commercial cellulase after moderate hydrothermal pretreatment. Artificially-constituted mixtures of leaf and stem tissues were found to require differing levels of enzymes. The authors concluded that the leaf:stem ratio is important when optimizing conversion processes and additionally in feedstock breeding. Our present study highlights the different ratios of leaf and stem within a wide range of wheat cultivars, thus indicating that there is significant potential for breeding wheat with varying tissue ratios.

## Conclusions

Using PLS models to rapidly quantify tissues and chemistry of straw from 90 cultivars has demonstrated a wide variation in chemistry which is strongly influenced by relative levels of tissues, particularly stem and leaf. Glucose, xylose and lignin positively correlate with stem proportion and height, but negatively correlate with leaf tissue. Pectins and diferulates positively correlate with leaf tissue but negatively correlate with stem and height. Total polysaccharide is also affected by the relative levels of non-carbohydrate components. Polysaccharides within tissues are highly conserved. The variation is likely to have significant impact on the potential to convert the biomass into biofuels or chemicals.

## Materials and Methods

### Wheat straw samples

#### Development of Fourier transform infrared models

Plants from six lines (cv Avalon, Cadenza, Charger, Paragon, Robigus and Savannah; four plants per line) were grown to maturity under greenhouse conditions at the John Innes Centre, Norwich. After harvest, the plants’ physical dimensions were measured and the grain and any ‘grain husks’ removed from the ear using a scalpel, leaving the spikelets. The remaining material was divided into four fractions: internode, node (including the true node and leaf base), leaf and ear. The leaves were connected at the node and wrapped tightly around the stem, often passing the next node along and completely enveloping it. Care had to be taken to remove all the external leaf that was wrapped around the stem. The plants were stored for two to three months in an ambient room temperature atmosphere to ensure air-dryness. Stems were cut at the ‘taper point’ above and below the nodes, to leave separate nodes and internodes. The share of these fractions in the total dry mass of the plant was determined gravimetrically.

#### Field samples

90 cultivars of wheat (listed in Table 3) were grown in the UK at KWS UK, Rothamsted Research and 17 Velcourt-managed farms, and harvested in the summer of 2010. All lines were re-sown at KWS to enable a second year of phenotyping, and material was harvested in the summer of 2011. The field-grown wheat plants were cut at the roots and dried in air at ambient conditions. The grain was separated from the ‘waste stream’ tissues and the whole straw used for analysis. For each cultivar, three plants were harvested. These were left to dry for two to three months to ensure air dryness before samples were milled to less than 250 μm prior to analysis.

**Table 3:** **Wheat cultivars used in the study**

ACCESS	COURTOT	HOLDFAST	MULIIWEISS	SOISSONS
ALBA	DEBEN	HUMBER	NAUTICA	SOLSTICE
ALBATROSS	EINSTEIN	HUSTLER	NORMAN	SPARK
ALCHEMY	EQUINOX	HYBRID-46	OAKLEY	SPERBER
AMBROSIA	ERLA-KOLBEN	HYPERION	OBELISK	STAMM 101
APACHE,USA	ESCORIAL	ISTABRAQ	ORLANDO	STARKE2
AVALON	ETOILE-DE-CHOISY	KAVKAZ	PALUR	STEADFAST
BACANORA	EXSEPT	KONTRAST	PARAGON	SVALE
BATTALION	EXTREM	LEDA	PERLO	TADORNA
BEAVER	FANAL	LONGBOW	PIKO	TARAS
BOREONOS	FLAIR	MALACCA	RABE,DEU	TREMIE
BUSTER	FLAME	MARCO	RECITAL	TRINTELLA
CALIF	FLORIDA	MARIS-HUNTSMAN	RENASANSA	TSCHERMAKS
CAPELLE-DESPREZ	GALAHAD	MARIS-WIDGEON	RIALTO	VILMORIN-27
CAPO	GATSBY	MEGA	RIBAND	VIRGO
CEZANNE	GLADIATOR	MENDEL	RIMPAUS-BRAUN	VIRTUE
CHARGER	GLASGOW	MERCIA	SAVANNAH	WEEBIL
CLAIRE	HAVEN	MIRAS	SCHWEIGERS-TACA	WERLA
CONSORT	HEREWARD	MIRONOVSKA	SHAMROCK	XI19
CORDIALE	HOBBIT	MUCK	SHANGO	ZEBEDEE

### Sample homogenisation by milling

Wheat straw is a heterogeneous and highly structured material. Because the applied analysis methods use only small amounts of material, the straw was homogenized in order to enable representative sampling. The equilibrated air-dry (between 7 and 8% moisture) wheat tissue fractions or whole plants were milled with a J&K MF10 analytical sieve mill (IKA®-Werke GmbH & Co. KG; Janke & Kunkel-Str. 10; Staufen, Germany) to less than 250 μm. Any remaining material greater than 250 μm was re-milled for 7 minutes with a J&K A10 grinder with a water cooling jacket (IKA®-Werke GmbH & Co. KG; Janke & Kunkel-Str. 10; Staufen, Germany) to less than 250 μm. The milled powder was mixed thoroughly before being measured by Fourier transform infrared (FTIR) spectroscopy and analyzed for chemical composition.

### Sugars analysis

Sugars were released from the fractions by hydrolysis with H_2_SO_4_ (72% w/w) for 3 hours at room temperature, followed by dilution to 1 mol L^−1^, and hydrolysis at 100°C for 2.5 hours (Saemen method of hydrolysis [9]). Hydrolyzed monosaccharides were analyzed as their alditol acetates by gas chromatography (GC) on a Perkin-Elmer (Waltham, Massachusetts, USA) Autosystem XL (GC1), Column: Restek Rtx-225, 30 m, 0.32 mm internal diameter (ID), 0.25 μm column film thickness (df), with flame ionization detection (FID) [19] using 2-deoxyglucose (200 μL, 1 mg mL^−1^) as an internal standard. Total uronic acid content was determined colorimetrically by the method of Blumenkrantz and Asboe-Hansen [20], using glucuronic acid as a standard. Each determination was carried out in triplicate.

### Phenolics analysis

Phenolic acids were extracted from the samples with progressively higher concentrations of alkali and quantified using HPLC with a Perkin-Elmer series 200 LC pump, Perkin-Elmer advanced LC Processor ISS200, Phenomenex Column Luna 5 μ C18(2), 250 × 4.0 mm with pre-column, and Perkin Elmer (Waltham, Massachusetts, USA) Diode Array Detector (UV) [21]. Analytical grade reagents and HPLC grade solvents were used.

### Lignin determination

Lignin was determined as 'Klason lignin' using the method described by Wood *et al*. [22] with the addition of sample stirring during the initial treatment with 72% sulfuric acid. Subsequently the sulphuric acid was diluted to 1 M and the polysaccharides were heated at 100°C to complete hydrolysis, leaving as a residue Klason lignin (a mixture of lignin, residual protein and ash).

### Fourier transform infrared attenuated total reflection (FTIR-ATR) spectroscopy

All FTIR was carried out using ATR sampling. FTIR-ATR spectra were measured with a BioRad FTS175 Fourier transform infrared spectrometer equipped with a MCT detector and a GoldenGate (Specac; Orpington, Kent, UK) single reflection diamond ATR accessory (BioRad, Cambridge, MA, USA). Five replicates from each milled sample powder were individually loaded on the ATR crystal and pressed down with the clamp. For each replicate, 64 spectra at 4 cm^−1^ resolution in the region of 4000 to 800 cm^−1^ were averaged and referenced against a spectrum of the empty crystal.

### Partial least squares models

The spectra were analyzed with MATLAB V7.14 (MathWorks Inc., Natick, Massachusetts, USA). The spectral range was truncated to 1800 to 800 cm^−1^ and any linear offset was removed by zeroing the absorption at 1800 cm^−1^. Additional baseline correction was performed with a fourth order polynomial anchored at the spectra minima. All spectra were area-normalized after baseline correction. First derivatives of the spectra were calculated with a three point moving window. PLS models for each variable were generated with the ‘plsregress’ function in the MATLAB statistics toolbox V8.0 (MathWorks Inc., Natick, Massachusetts, USA). Internal 'leave one out' cross-validation was used, both for individual samples and blocks of samples from whole wheat lines. The optimal numbers of PLS factors for the individual models were determined from the percentage of explained variation and residual errors.

### Partial least squares model of chemical composition

PLS models were generated for total sugar content, and separately for the contents of glucose, rhamnose, fucose, arabinose, xylose, mannose and galactose, as well as uronic acid.

PLS models were made for contents of lignin, acid insoluble ash, and acid insoluble ash corrected lignin.

PLS models were made for the contents of protocatechuic acid, protocatechuic aldehyde, chlorogenic acid, p-OH-benzoic acid, p-OH-phenyl acetic acid, vanillic acid, caffeic acid, p-OH-benzaldehyde, truxillic acid (coumaric acid), truxillic acid (ferulic acid), vanillin, trans-p-coumaric acid 8,8'-DiFA (aryl tetralin), sinapic acid, FA, cis-p-coumaric acid, 8,8'-DiFA, 8,5'-DiFA, cis-ferulic acid, 5,5'-DiFA, 8-O-4'-DiFA, 8,5'-DiFA (benzofuran), and total phenolics (including unknown peaks).

The calibration set was chosen to deal with the wide variety of sample parameters (Table 1). The set contained whole-plant samples from eight field-grown wheat lines (one plant each of cv Consort, Deben, Etoile-de-Choisy, Extrem, Hustler, Orlando, Sperber and Steadfast) as well as samples from six greenhouse-grown wheat lines (four plants each of cvs Avalon, Cadenza, Charger, Paragon, Robigus and Savannah) which had been dissected into internode, node, leaf and ear. For these six lines, the chemical compositions and spectra of the whole-plant samples were calculated from the measurements of the individual tissues and their percentage dry weight. By using separated tissues, a wider range of concentrations was available for calibrating the PLS models, as was suggested in earlier studies [6,10]. In tissues, the total sugars content ranged from 425 to 629 mg/g, compared with 463 to 574 in whole plants.

In order to account for ATR sampling variations, all five replicate spectra from each reference sample were fitted individually against the reference values. The replicate field line sample spectra were subjected individually to the PLS analysis, and their PLS prediction results averaged at the end.

### PLS model of tissue composition

In order to estimate the distribution of tissues within biomass samples from field samples, a PLS model was derived from the FTIR spectra and air-dried biomass weights of the four tissues (node, internode, leaf and ear) from each of four replicate plants from the six greenhouse-grown lines (cv Avalon, Cadenza, Charger, Paragon, Robigus and Savannah). The tissue spectra from individual plants were convoluted and fitted to the measured amounts (in percentage weight) of the four tissues in the whole plants. Spectra from the individual tissues were modelled as 100% of the respective tissue type.

## Additional files

Additional file 1: Table S1. Carbohydrate and lignin composition of component tissues of wheat straw cultivars. Additional file 2: Table S2. Phenolic compositions of the component tissues from six wheat cultivars. Additional file 3: Table S3. Published data on composition of whole wheat straw and component tissues. Additional file 4: Table S4. Correlation table for 2011.

## Abbreviations

AVA: Avalon
CAD: Cadenza
CHA: Charger
DM: Dry matter
FA: *Trans*-Ferulic Acid
FTIR: Fourier transform infra red
FT-NIR: Fourier transform near infrared
GC: Gas chromatography
PAR: Paragon
*p*CA: *Para*-Coumaric Acid
PLS: Partial Least Squares
RMSEC: Root mean square error of calibration
ROB: Robigus
SAV: Savanah
