# Supplementary material for: Variation in the chemical composition of wheat straw: the role of tissue ratio and composition
Source: Biotechnol Biofuels. 2014 Aug 20;7:121. doi: 10.1186/s13068-014-0121-y (PMC4243778; doi:10.1186/s13068-014-0121-y)
Supplement: Additional file 1: Table S1. — Carbohydrate and lignin composition of component tissues of wheat straw cultivars. [file 13068_2014_121_MOESM1_ESM.docx]

**Table S1.** Carbohydrate and lignin composition of component tissues of wheat straw cultivars

|  | **Total sugars** |  | **Rha** | | **Fuc** | | **Ara** | | **Xyl** | | **Man** | | **Gal** | | **Glc** | | **UA** | | **Cor. Lignin** | |
| --- | --- | --- | --- | --- | --- | --- | --- | --- | --- | --- | --- | --- | --- | --- | --- | --- | --- | --- | --- | --- |
|  | **mean** | **s.d.** | **mean** | **s.d.** | **mean** | **s.d.** | **mean** | **s.d.** | **mean** | **s.d.** | **mean** | **s.d.** | **mean** | **s.d.** | **mean** | **s.d.** | **mean** | **s.d.** | **mean** | **s.d.** |
| **CADENZA** | |  |  |  |  |  |  |  |  |  |  |  |  |  |  |  |  |  |  |  |
| **Internode** | 691.54 | 24.15 | 0.76 | 0.05 | 0.11 | 0.02 | 16.00 | 0.84 | 187.81 | 10.05 | 8.30 | 5.45 | 4.32 | 0.21 | 441.83 | 20.62 | 32.41 | 5.29 | 184.69 | 15.78 |
| **Node** | 655.08 | 30.70 | 1.44 | 0.19 | 0.27 | 0.04 | 38.23 | 1.96 | 171.13 | 16.95 | 9.80 | 4.03 | 7.15 | 3.91 | 379.42 | 14.98 | 47.64 | 5.15 | 145.98 | 16.56 |
| **Leaf** | 636.89 | 88.78 | 1.83 | 0.54 | 0.42 | 0.11 | 30.46 | 1.47 | 192.01 | 42.48 | 3.58 | 1.68 | 9.09 | 4.89 | 353.04 | 46.48 | 46.47 | 2.15 | 166.82 | 11.12 |
| **Ear** | 649.31 | 49.85 | 1.25 | 0.52 | 0.30 | 0.14 | 29.25 | 2.76 | 211.99 | 27.25 | 2.21 | 0.80 | 10.07 | 1.44 | 366.88 | 28.13 | 27.36 | 6.45 | 150.88 | 7.65 |
| **PARAGON** | |  |  |  |  |  |  |  |  |  |  |  |  |  |  |  |  |  |  |  |
| **Internode** | 561.24 | 18.53 | 0.70 | 0.26 | 0.14 | 0.05 | 13.66 | 1.13 | 150.95 | 12.42 | 18.25 | 5.84 | 3.61 | 0.61 | 349.02 | 12.02 | 24.91 | 3.70 | 185.33 | 20.04 |
| **Node** | 557.07 | 25.80 | 0.87 | 0.27 | 0.24 | 0.10 | 30.33 | 11.20 | 151.30 | 11.52 | 15.82 | 4.75 | 6.90 | 2.15 | 317.83 | 34.84 | 33.79 | 4.96 | 167.47 | 11.03 |
| **Leaf** | 538.02 | 23.41 | 1.35 | 0.41 | 0.38 | 0.09 | 30.62 | 3.48 | 166.09 | 9.68 | 9.82 | 5.42 | 9.43 | 1.04 | 284.80 | 15.86 | 35.52 | 3.99 | 171.26 | 16.23 |
| **Ear** | 575.33 | 37.01 | 0.89 | 0.47 | 0.25 | 0.10 | 29.65 | 0.86 | 205.03 | 30.34 | 4.64 | 2.71 | 9.52 | 1.52 | 298.56 | 18.01 | 26.79 | 7.57 | 157.58 | 5.74 |
| **SAVANAH** | |  |  |  |  |  |  |  |  |  |  |  |  |  |  |  |  |  |  |  |
| **Internode** | 557.63 | 15.00 | 0.83 | 0.13 | 0.16 | 0.04 | 14.15 | 0.91 | 147.15 | 11.79 | 13.31 | 4.86 | 3.85 | 0.33 | 353.96 | 11.52 | 24.22 | 1.49 | 151.83 | 17.76 |
| **Node** | 537.81 | 25.67 | 1.05 | 0.09 | 0.26 | 0.03 | 33.33 | 4.08 | 133.82 | 13.81 | 15.58 | 4.94 | 7.77 | 0.87 | 312.86 | 9.43 | 33.15 | 3.69 | 124.25 | 12.69 |
| **Leaf** | 535.96 | 20.65 | 1.43 | 0.73 | 0.49 | 0.04 | 30.14 | 1.37 | 159.91 | 9.83 | 5.73 | 0.91 | 11.89 | 0.38 | 291.36 | 13.40 | 35.01 | 3.63 | 148.12 | 6.31 |
| **Ear** | 620.51 | 15.65 | 0.70 | 0.10 | 0.30 | 0.05 | 30.82 | 0.69 | 209.08 | 3.77 | 2.61 | 0.29 | 9.59 | 0.28 | 343.37 | 12.05 | 24.03 | 1.53 | 138.36 | 8.74 |
| **ROBIGUS** | |  |  |  |  |  |  |  |  |  |  |  |  |  |  |  |  |  |  |  |
| **Internode** | 559.61 | 15.12 | 0.86 | 0.11 | 0.41 | 0.11 | 14.92 | 0.45 | 149.21 | 7.89 | 6.65 | 4.25 | 3.76 | 0.30 | 351.35 | 7.19 | 32.46 | 2.30 | 205.02 | 14.71 |
| **Node** | 512.21 | 35.62 | 1.19 | 0.16 | 0.61 | 0.05 | 33.45 | 2.96 | 138.31 | 10.74 | 8.56 | 1.77 | 6.88 | 0.51 | 276.82 | 24.35 | 46.38 | 3.00 | 186.94 | 11.42 |
| **Leaf** | 520.13 | 7.35 | 2.16 | 0.12 | 1.10 | 0.59 | 28.73 | 0.64 | 143.62 | 5.16 | 3.74 | 0.59 | 9.57 | 0.35 | 279.83 | 3.42 | 51.39 | 2.50 | 148.70 | 7.09 |
| **Ear** | 586.49 | 9.72 | 1.05 | 0.26 | 0.59 | 0.20 | 30.02 | 1.33 | 205.09 | 3.82 | 2.41 | 0.19 | 9.43 | 0.57 | 308.25 | 10.84 | 29.65 | 1.29 | 162.15 | 6.01 |
| **CHARGER** | |  |  |  |  |  |  |  |  |  |  |  |  |  |  |  |  |  |  |  |
| **Internode** | 542.18 | 20.14 | 0.70 | 0.18 | 0.37 | 0.06 | 14.94 | 0.86 | 159.45 | 11.26 | 12.12 | 3.73 | 3.60 | 0.51 | 325.94 | 11.38 | 25.06 | 1.73 | 172.26 | 7.51 |
| **Node** | 538.07 | 23.44 | 1.10 | 0.08 | 0.48 | 0.08 | 36.47 | 3.82 | 154.46 | 15.12 | 12.30 | 3.47 | 7.89 | 0.85 | 289.86 | 10.40 | 35.50 | 4.79 | 137.66 | 5.97 |
| **Leaf** | 458.94 | 35.56 | 1.57 | 0.29 | 0.71 | 0.07 | 26.52 | 3.40 | 137.67 | 18.00 | 5.68 | 0.75 | 10.14 | 0.84 | 240.96 | 13.63 | 35.71 | 3.86 | 151.61 | 5.92 |
| **Ear** | 567.83 | 12.27 | 0.70 | 0.09 | 0.53 | 0.08 | 31.90 | 0.37 | 207.48 | 10.93 | 3.05 | 0.47 | 9.43 | 0.28 | 290.32 | 14.21 | 24.41 | 1.74 | 149.67 | 3.02 |
| **AVALON** | |  |  |  |  |  |  |  |  |  |  |  |  |  |  |  |  |  |  |  |
| **Internode** | 501.62 | 30.10 | 0.63 | 0.20 | 1.09 | 0.25 | 14.03 | 2.17 | 140.42 | 31.18 | 25.83 | 15.44 | 3.33 | 0.41 | 290.52 | 12.08 | 25.75 | 2.78 | 134.57 | 24.50 |
| **Node** | 505.54 | 27.46 | 0.85 | 0.11 | 1.05 | 0.21 | 30.58 | 8.31 | 132.73 | 30.45 | 26.81 | 13.09 | 6.57 | 1.43 | 273.91 | 6.67 | 33.03 | 2.05 | 109.29 | 18.92 |
| **Leaf** | 462.86 | 41.60 | 1.52 | 0.24 | 1.26 | 0.31 | 27.95 | 2.63 | 146.61 | 23.52 | 9.39 | 5.03 | 9.87 | 0.57 | 229.14 | 19.01 | 37.13 | 3.49 | 136.39 | 11.23 |
| **Ear** | 538.95 | 42.58 | 0.62 | 0.14 | 0.95 | 0.11 | 29.53 | 1.58 | 205.42 | 9.10 | 3.33 | 0.96 | 8.29 | 0.53 | 263.99 | 28.82 | 26.82 | 6.35 | 134.04 | 8.36 |
