# Supplementary material for: Variation in the chemical composition of wheat straw: the role of tissue ratio and composition
Source: Biotechnol Biofuels. 2014 Aug 20;7:121. doi: 10.1186/s13068-014-0121-y (PMC4243778; doi:10.1186/s13068-014-0121-y)
Supplement: Additional file 2: Table S2. — Phenolic compositions of the component tissues from six wheat cultivars. [file 13068_2014_121_MOESM2_ESM.docx]

***Table S2****: Phenolic compositions of the component tissues from six wheat cultivars*

|  | **Protocatechuic Aldehyde** | | **p-OH-benzoic acid** | | **Vanillic acid** | | **p-OH-benzaldehyde** | | **Truxillic Acid (CA)** | | **Truxillic Acid (FA)** | | **Vanillin** | | **trans-p-coumaric acid** | | **trans-ferulic acid** | | **8,8' -DiFA** | | **8,5'-DiFA** | | **5,5'-DiFA** | | **8-O-4'-DiFA** | | **8,5'-DiFA (benzofuran)** | |
| --- | --- | --- | --- | --- | --- | --- | --- | --- | --- | --- | --- | --- | --- | --- | --- | --- | --- | --- | --- | --- | --- | --- | --- | --- | --- | --- | --- | --- |
|  | **Mean** | **SE** | **Mean** | **SE** | **Mean** | **SE** | **Mean** | **SE** | **Mean** | **SE** | **Mean** | **SE** | **Mean** | **SE** | **Mean** | **SE** | **Mean** | **SE** | **Mean** | **SE** | **Mean** | **SE** | **Mean** | **SE** | **Mean** | **SE** | **Mean** | **SE** |
| **PARAGON** |  |  |  |  |  |  |  |  |  |  |  |  |  |  |  |  |  |  |  |  |  |  |  |  |  |  |  |  |
| **Internode** | 0.005 | 0.001 | 0.029 | 0.008 | 0.082 | 0.013 | 0.021 | 0.005 | 0.003 | 0.001 | 0.014 | 0.005 | 0.055 | 0.011 | 2.660 | 0.704 | 1.819 | 0.499 | 0.025 | 0.008 | 0.082 | 0.044 | 0.059 | 0.016 | 0.195 | 0.056 | 0.075 | 0.017 |
| **Node** | 0.008 | 0.002 | 0.041 | 0.009 | 0.093 | 0.020 | 0.053 | 0.019 | 0.007 | 0.002 | 0.019 | 0.004 | 0.093 | 0.027 | 5.148 | 1.353 | 2.639 | 0.545 | 0.038 | 0.013 | 0.125 | 0.034 | 0.139 | 0.039 | 0.377 | 0.122 | 0.112 | 0.020 |
| **Leaf** | 0.009 | 0.002 | 0.049 | 0.012 | 0.148 | 0.020 | 0.036 | 0.004 | 0.010 | 0.003 | 0.033 | 0.007 | 0.156 | 0.049 | 3.254 | 0.928 | 2.936 | 0.196 | 0.073 | 0.020 | 0.432 | 0.132 | 0.179 | 0.024 | 0.611 | 0.090 | 0.178 | 0.032 |
| **Ear** | 0.006 | 0.001 | 0.044 | 0.012 | 0.105 | 0.017 | 0.049 | 0.014 | 0.007 | 0.001 | 0.023 | 0.004 | 0.130 | 0.027 | 3.680 | 1.114 | 2.759 | 0.522 | 0.051 | 0.010 | 0.270 | 0.070 | 0.241 | 0.043 | 0.606 | 0.089 | 0.152 | 0.033 |
| **AVALON** |  |  |  |  |  |  |  |  |  |  |  |  |  |  |  |  |  |  |  |  |  |  |  |  |  |  |  |  |
| **Internode** | 0.004 | 0.001 | 0.250 | 0.036 | 0.224 | 0.033 | 0.100 | 0.026 | 0.010 | 0.003 | 0.017 | 0.001 | 0.240 | 0.072 | 8.758 | 1.098 | 4.919 | 0.612 | 0.041 | 0.018 | 0.148 | 0.039 | 0.100 | 0.005 | 0.401 | 0.074 | 0.126 | 0.017 |
| **Node** | 0.005 | 0.001 | 0.234 | 0.051 | 0.199 | 0.028 | 0.106 | 0.036 | 0.010 | 0.002 | 0.017 | 0.001 | 0.159 | 0.058 | 10.531 | 1.425 | 5.130 | 0.454 | 0.054 | 0.014 | 0.215 | 0.046 | 0.206 | 0.038 | 0.636 | 0.150 | 0.197 | 0.040 |
| **Leaf** | 0.007 | 0.001 | 0.145 | 0.031 | 0.239 | 0.018 | 0.078 | 0.004 | 0.015 | 0.001 | 0.017 | 0.001 | 0.346 | 0.028 | 4.884 | 0.250 | 5.633 | 0.407 | 0.119 | 0.010 | 0.652 | 0.043 | 0.231 | 0.016 | 0.760 | 0.053 | 0.174 | 0.021 |
| **Ear** | 0.007 | 0.000 | 0.255 | 0.053 | 0.210 | 0.014 | 0.156 | 0.007 | 0.012 | 0.002 | 0.020 | 0.001 | 0.357 | 0.014 | 10.194 | 0.462 | 6.485 | 0.257 | 0.091 | 0.004 | 0.429 | 0.025 | 0.343 | 0.005 | 0.891 | 0.025 | 0.167 | 0.008 |
| **CHARGER** |  |  |  |  |  |  |  |  |  |  |  |  |  |  |  |  |  |  |  |  |  |  |  |  |  |  |  |  |
| **Internode** | 0.003 | 0.001 | 0.080 | 0.017 | 0.201 | 0.004 | 0.103 | 0.021 | 0.009 | 0.000 | 0.015 | 0.000 | 0.236 | 0.029 | 9.476 | 0.832 | 5.455 | 0.264 | 0.055 | 0.020 | 0.277 | 0.061 | 0.133 | 0.012 | 0.382 | 0.033 | 0.127 | 0.011 |
| **Node** | 0.004 | 0.001 | 0.135 | 0.032 | 0.154 | 0.005 | 0.137 | 0.027 | 0.007 | 0.001 | 0.015 | 0.001 | 0.204 | 0.035 | 12.215 | 1.096 | 5.195 | 0.386 | 0.040 | 0.014 | 0.288 | 0.051 | 0.186 | 0.020 | 0.526 | 0.064 | 0.134 | 0.016 |
| **Leaf** | 0.008 | 0.001 | 0.074 | 0.011 | 0.263 | 0.033 | 0.086 | 0.005 | 0.015 | 0.002 | 0.015 | 0.001 | 0.343 | 0.018 | 5.029 | 0.152 | 5.351 | 0.270 | 0.078 | 0.002 | 0.708 | 0.048 | 0.229 | 0.013 | 0.748 | 0.036 | 0.161 | 0.008 |
| **Ear** | 0.006 | 0.000 | 0.130 | 0.007 | 0.176 | 0.003 | 0.151 | 0.013 | 0.013 | 0.001 | 0.022 | 0.001 | 0.336 | 0.025 | 11.065 | 0.249 | 6.103 | 0.284 | 0.070 | 0.012 | 0.522 | 0.052 | 0.378 | 0.038 | 0.932 | 0.009 | 0.173 | 0.005 |
| **ROBIGUS** |  |  |  |  |  |  |  |  |  |  |  |  |  |  |  |  |  |  |  |  |  |  |  |  |  |  |  |  |
| **Internode** | 0.007 | 0.001 | 0.147 | 0.014 | 0.310 | 0.039 | 0.223 | 0.017 | 0.013 | 0.001 | 0.020 | 0.002 | 0.347 | 0.025 | 14.606 | 0.626 | 6.296 | 0.242 | 0.093 | 0.007 | 0.373 | 0.015 | 0.109 | 0.004 | 0.448 | 0.012 | 0.159 | 0.002 |
| **Node** | 0.011 | 0.004 | 0.277 | 0.110 | 0.271 | 0.036 | 0.269 | 0.034 | 0.016 | 0.004 | 0.022 | 0.005 | 0.296 | 0.027 | 17.868 | 1.003 | 6.368 | 0.038 | 0.099 | 0.010 | 0.479 | 0.091 | 0.172 | 0.002 | 0.668 | 0.065 | 0.196 | 0.021 |
| **Leaf** | 0.010 | 0.001 | 0.077 | 0.006 | 0.315 | 0.007 | 0.078 | 0.000 | 0.016 | 0.001 | 0.022 | 0.001 | 0.329 | 0.014 | 5.171 | 0.249 | 5.909 | 0.120 | 0.133 | 0.007 | 0.870 | 0.034 | 0.203 | 0.008 | 0.825 | 0.016 | 0.198 | 0.004 |
| **Ear** | 0.006 | 0.000 | 0.115 | 0.006 | 0.222 | 0.006 | 0.177 | 0.022 | 0.016 | 0.001 | 0.021 | 0.001 | 0.319 | 0.006 | 12.099 | 0.434 | 5.696 | 0.110 | 0.091 | 0.005 | 0.564 | 0.008 | 0.254 | 0.007 | 0.817 | 0.019 | 0.176 | 0.004 |
| **SAVANNAH** | |  |  |  |  |  |  |  |  |  |  |  |  |  |  |  |  |  |  |  |  |  |  |  |  |  |  |  |
| **Internode** | 0.007 | 0.001 | 0.033 | 0.004 | 0.268 | 0.021 | 0.053 | 0.014 | 0.000 | 0.000 | 0.022 | 0.002 | 0.085 | 0.011 | 4.611 | 0.644 | 3.251 | 0.220 | 0.075 | 0.010 | 0.293 | 0.030 | 0.110 | 0.009 | 0.855 | 0.503 | 0.176 | 0.022 |
| **Node** | 0.005 | 0.001 | 0.041 | 0.006 | 0.208 | 0.032 | 0.069 | 0.016 | 0.000 | 0.000 | 0.021 | 0.001 | 0.067 | 0.007 | 5.539 | 0.646 | 2.851 | 0.130 | 0.062 | 0.010 | 0.304 | 0.027 | 0.169 | 0.010 | 0.523 | 0.042 | 0.151 | 0.013 |
| **Leaf** | 0.014 | 0.003 | 0.030 | 0.002 | 0.365 | 0.032 | 0.040 | 0.001 | 0.000 | 0.000 | 0.036 | 0.002 | 0.153 | 0.017 | 2.541 | 0.130 | 4.000 | 0.407 | 0.098 | 0.011 | 0.995 | 0.065 | 0.234 | 0.018 | 0.996 | 0.063 | 0.286 | 0.042 |
| **Ear** | 0.013 | 0.006 | 0.059 | 0.001 | 0.237 | 0.004 | 0.082 | 0.007 | 0.000 | 0.000 | 0.023 | 0.002 | 0.109 | 0.008 | 4.954 | 0.428 | 3.382 | 0.088 | 0.084 | 0.005 | 0.626 | 0.037 | 0.321 | 0.022 | 0.892 | 0.040 | 0.176 | 0.012 |
| **CADENZA** |  |  |  |  |  |  |  |  |  |  |  |  |  |  |  |  |  |  |  |  |  |  |  |  |  |  |  |  |
| **Internode** | 0.009 | 0.001 | 0.075 | 0.011 | 0.167 | 0.012 | 0.096 | 0.006 | 0.019 | 0.000 | 0.021 | 0.001 | 0.222 | 0.017 | 7.680 | 0.345 | 4.269 | 0.127 | 0.120 | 0.003 | 0.403 | 0.019 | 0.119 | 0.009 | 0.540 | 0.014 | 0.187 | 0.011 |
| **Node** | 0.007 | 0.000 | 0.095 | 0.007 | 0.117 | 0.008 | 0.116 | 0.007 | 0.015 | 0.001 | 0.017 | 0.001 | 0.155 | 0.012 | 8.013 | 0.360 | 3.352 | 0.113 | 0.080 | 0.002 | 0.391 | 0.022 | 0.163 | 0.009 | 0.559 | 0.019 | 0.142 | 0.002 |
| **Leaf** | 0.010 | 0.001 | 0.048 | 0.004 | 0.176 | 0.003 | 0.055 | 0.003 | 0.020 | 0.001 | 0.020 | 0.001 | 0.248 | 0.009 | 3.634 | 0.030 | 3.865 | 0.020 | 0.118 | 0.004 | 0.926 | 0.013 | 0.251 | 0.004 | 0.983 | 0.023 | 0.207 | 0.002 |
| **Ear** | 0.008 | 0.000 | 0.052 | 0.001 | 0.152 | 0.006 | 0.077 | 0.005 | 0.019 | 0.001 | 0.017 | 0.000 | 0.244 | 0.013 | 5.147 | 0.329 | 3.804 | 0.180 | 0.104 | 0.007 | 0.773 | 0.026 | 0.278 | 0.008 | 0.903 | 0.038 | 0.173 | 0.003 |
