# Supplementary material for: Variation in the chemical composition of wheat straw: the role of tissue ratio and composition
Source: Biotechnol Biofuels. 2014 Aug 20;7:121. doi: 10.1186/s13068-014-0121-y (PMC4243778; doi:10.1186/s13068-014-0121-y)
Supplement: Additional file 3: Table S3. — Published data on composition of whole wheat straw and component tissues. [file 13068_2014_121_MOESM3_ESM.docx]

**Tabls S3.** Published data on composition of whole wheat straw and component tissues

| **Reference** | **Material** | **Tissue%** | **Ara** | **Xyl** | **Glc** | **Lig** | **Hem** |
| --- | --- | --- | --- | --- | --- | --- | --- |
|  |  |  |  |  |  |  |  |
| Heiss-Blanquet et al 2011 | WWS |  |  |  | 39.3 | 16.2 | 20.7 |
| Thomsen et al (2008) | WWS |  | 2.7 | 19.7 | 33.5 | 16.4 | 22.4 |
| Kaparaju et al (2009) | WWS |  | 2.6 | 21.3 | 35.9 | 19.3 | n/a |
| Ballesteros et al (2006) | WWS |  | 2.8 | 18.7 | 30.2 | 15.3 | 22.3 |
| Linde et al (2008) | WWS |  | 3.3 | 20.1 | 32.6 | 24.2 | 24.2 |
| Harper and Lynch (1981) | WS cv Huntsman Internode | |  |  | 44.8 | 14.2 | 33.8 |
| Harper and Lynch (1981) | WS cv Huntsman leaf |  |  |  | 37.7 | 15.3 | 32.4 |
| Harper and Lynch (1981) | WS cv Huntsman node |  |  |  | 37.5 | 16.7 | 32.7 |
| Harper and Lynch (1981) | WS cv Huntsman leaf base | |  |  | 32.7 | 14.1 | 34.2 |
| Jacobs et al (on the web) (circa 2000) | WS cv Madsen internode | 53.2 | 1.7 | 23.6 | 42 | 19.3 | 25.9 |
| Jacobs et al (on the web) (circa 2000) | WS cv Madsen leaf | 37.7 | 3.2 | 21.4 | 29.6 | 14 | 26.1 |
| Jacobs et al (on the web) (circa 2000) | WS cv Madsen node | 9.1 | 4.2 | 21.2 | 34.5 | 18.8 | 26.9 |
| Lomborg et al (2010) | WS no cv data min values |  | 2.1 | 18.9 | 34.9 | 18.8 |  |
| Lomborg et al (2010) | WS no cv data max values |  | 3.1 | 22.8 | 40.3 | 24.6 |  |
| Tamaki and Mazza (2011) | WWS range of cvs |  |  |  |  | 15.94 |  |
| Lindedam et al (2010) | WWS mean from range of cvs | |  |  | 36.6 | 19.5 | 25.6 |
| Lindedam et al (2010) | WWS Min value |  |  |  | 34.2 | 18.2 | 23.2 |
| Lindedam et al (2010) | WWS Max value |  |  |  | 40.8 | 20.4 | 28.6 |
| Pronyk and Mazza (2012) | WWS Durum wheat |  | 1.64 | 19.68 | 39.42 | 18.15 |  |
| Pronyk and Mazza (2012) | WWS CPS |  | 1.82 | 19.3 | 37.88 | 18.38 |  |
| Lindedam et al 2012 | WWS cvSkalmeje |  |  |  | 34.4 |  | 23.5 |
| Lindedam et al 2013 | WWS cvInspiration |  |  |  | 39.7 |  | 26.9 |
