# Supplementary material for: Variation in the chemical composition of wheat straw: the role of tissue ratio and composition
Source: Biotechnol Biofuels. 2014 Aug 20;7:121. doi: 10.1186/s13068-014-0121-y (PMC4243778; doi:10.1186/s13068-014-0121-y)
Supplement: Additional file 4: Table S4. — Correlation table for 2011. [file 13068_2014_121_MOESM4_ESM.pdf]

TABLE S4: CORRELATION TABLE FOR 2011

score

	height	steml	width	tillers	totwt	wtear	earwt	smax	fmax	d2	i	moe	mor	xylose	rhamnose	mannose	lignin	glucose	galactose	fucose	t_ferulic
height																					
steml	0.993																				
width	0.001	-0.009																			
tillers	-0.004	-0.011	-0.062																		
totwt	0.146	0.124	-0.048	0.797																	
wtear	0.609	0.588	-0.075	0.529	0.715																
earwt	-0.135	-0.152	-0.022	0.761	0.927	0.402															
smax	0.516	0.497	0.006	-0.091	0.092	0.312	-0.047														
fmax	0.131	0.124	-0.018	-0.019	0.057	0.047	0.049	0.012													
d2	-0.097	-0.092	0.019	-0.053	-0.071	-0.165	-0.005	-0.446	0.809												
i	0.037	0.041	0.060	-0.235	-0.200	-0.193	-0.159	0.061	0.029	0.293											
moe	0.194	0.188	-0.081	0.140	0.173	0.282	0.075	0.446	-0.165	-0.556	-0.599										
mor	0.114	0.101	-0.110	0.069	0.147	0.185	0.093	0.259	0.680	0.241	-0.369	0.444									
xylose	0.338	0.353	0.099	-0.340	-0.278	-0.134	-0.290	0.211	0.220	0.173	0.141	-0.011	0.072								
rhamnose	-0.282	-0.294	-0.084	0.426	0.227	0.042	0.268	-0.100	-0.267	-0.258	-0.207	0.161	-0.043	-0.529							
mannose	-0.117	-0.127	-0.061	-0.003	0.015	0.099	-0.026	-0.057	-0.214	-0.200	-0.147	0.056	-0.088	-0.717	0.074						
lignin	0.372	0.389	-0.041	-0.237	-0.336	-0.064	-0.394	0.149	0.098	0.093	0.120	-0.021	0.009	0.727	-0.148	-0.586					
glucose	0.637	0.629	0.129	-0.079	-0.012	0.260	-0.132	0.344	0.132	0.023	0.118	0.101	0.059	0.397	-0.230	-0.303	0.486				
galactose	-0.671	-0.674	-0.104	0.291	0.175	-0.132	0.279	-0.384	-0.272	-0.173	-0.275	0.050	-0.054	-0.695	0.609	0.505	-0.568	-0.667			
fucose	-0.393	-0.391	-0.069	-0.139	-0.054	-0.332	0.081	-0.132	0.042	0.068	0.043	-0.097	0.014	-0.035	-0.031	-0.061	-0.315	-0.642	0.165		
t_ferulic	-0.247	-0.256	0.014	-0.031	0.067	-0.213	0.179	0.025	0.114	0.031	-0.024	0.036	0.124	0.009	0.109	-0.308	-0.264	-0.318	-0.028	0.753	
p_acetic	0.331	0.327	0.041	-0.200	-0.226	0.013	-0.290	0.436	0.118	-0.056	0.090	0.162	0.129	0.406	0.012	-0.289	0.445	0.434	-0.469	-0.172	0.060
benzoic	-0.210	-0.210	-0.052	-0.327	-0.191	-0.331	-0.092	-0.083	0.092	0.088	-0.012	-0.065	0.062	0.201	-0.250	-0.133	-0.039	-0.344	-0.043	0.780	0.654
c_coumaric	-0.054	-0.059	0.043	-0.023	-0.069	-0.107	-0.039	0.097	0.116	0.028	-0.131	0.071	0.145	0.138	0.309	-0.295	0.219	-0.105	-0.050	0.342	0.558
benzaldehyde	-0.322	-0.308	-0.075	-0.202	-0.111	-0.362	0.023	-0.166	0.155	0.160	0.092	-0.122	0.114	0.208	-0.124	-0.380	0.078	-0.295	-0.013	0.643	0.695
c_ferulic	-0.377	-0.364	-0.134	0.008	0.031	-0.204	0.130	-0.325	-0.200	-0.050	-0.040	-0.029	-0.109	-0.129	0.083	-0.016	-0.229	-0.343	0.383	0.388	0.116
vanillic	-0.322	-0.325	-0.092	0.169	0.270	-0.015	0.347	-0.337	-0.016	0.079	-0.060	-0.095	-0.031	-0.428	-0.110	0.236	-0.596	-0.199	0.313	0.207	0.256
trux_FA	-0.047	-0.052	-0.068	0.319	0.332	0.241	0.310	-0.235	-0.159	-0.069	-0.123	0.013	-0.112	-0.528	0.054	0.416	-0.489	0.065	0.311	-0.324	-0.293
trux_CA	0.134	0.126	0.047	-0.181	-0.179	-0.101	-0.179	0.277	0.171	0.051	0.061	0.090	0.177	0.460	0.106	-0.578	0.518	0.163	-0.391	0.249	0.479
totp_unknown	-0.274	-0.278	-0.010	-0.075	0.047	-0.245	0.169	-0.017	0.132	0.068	0.028	0.008	0.142	0.074	0.037	-0.379	-0.178	-0.305	-0.030	0.743	0.953
totsugars	0.574	0.566	0.172	-0.131	-0.105	0.151	-0.201	0.347	0.153	0.046	0.124	0.084	0.066	0.549	-0.186	-0.463	0.631	0.938	-0.703	-0.528	-0.193
totphenolics	-0.282	-0.285	-0.015	-0.087	0.037	-0.255	0.162	-0.029	0.131	0.073	0.031	0.000	0.135	0.076	0.016	-0.367	-0.177	-0.320	-0.028	0.761	0.950
proto_aldehyde	-0.224	-0.228	-0.160	0.272	0.245	0.138	0.246	-0.336	-0.153	-0.008	-0.044	-0.055	-0.101	-0.551	0.090	0.434	-0.396	-0.167	0.444	-0.088	-0.253
corr_lignin	0.442	0.461	-0.042	-0.187	-0.268	0.016	-0.344	0.172	0.109	0.112	0.179	-0.028	0.000	0.739	-0.212	-0.630	0.973	0.547	-0.646	-0.382	-0.307
ash	-0.151	-0.152	-0.010	-0.280	-0.367	-0.298	-0.329	-0.007	-0.039	-0.095	-0.204	0.049	0.037	0.051	0.216	0.160	0.278	-0.080	0.172	0.133	0.056
d804	-0.489	-0.506	0.036	0.256	0.340	-0.088	0.468	-0.246	-0.092	-0.068	-0.116	0.030	-0.001	-0.583	0.280	0.212	-0.764	-0.346	0.507	0.445	0.556
d88	0.071	0.058	-0.005	0.074	0.217	0.071	0.241	-0.004	0.024	0.015	-0.057	0.046	0.015	-0.032	0.018	-0.164	-0.222	0.323	-0.130	0.218	0.458
benzofuran	-0.167	-0.184	0.021	0.202	0.350	0.125	0.385	-0.183	0.002	0.049	-0.035	-0.026	-0.012	-0.460	-0.103	0.251	-0.674	-0.037	0.150	0.214	0.319
d85	-0.150	-0.158	-0.125	0.177	0.285	0.057	0.333	-0.181	-0.055	0.009	-0.072	0.066	-0.022	-0.233	0.032	-0.048	-0.275	0.121	0.111	0.191	0.318
d55	-0.540	-0.544	0.063	-0.060	-0.042	-0.389	0.122	-0.238	-0.122	-0.107	-0.155	0.009	-0.040	-0.279	0.279	0.127	-0.338	-0.571	0.471	0.698	0.618
node	-0.090	-0.090	0.007	0.033	0.113	0.082	0.106	-0.236	0.052	0.167	0.076	-0.153	0.003	-0.136	-0.371	0.161	-0.136	0.008	0.087	-0.193	-0.295
leaf	-0.630	-0.629	-0.139	0.260	0.109	-0.199	0.228	-0.268	-0.241	-0.174	-0.100	0.054	-0.041	-0.638	0.710	0.334	-0.479	-0.641	0.888	0.222	0.072
inter	0.757	0.758	0.025	-0.208	-0.134	0.226	-0.272	0.390	0.117	-0.029	0.036	0.110	0.047	0.489	-0.364	-0.106	0.488	0.637	-0.705	-0.411	-0.219
ear	0.035	0.033	0.168	-0.111	0.014	0.037	0.001	-0.070	0.201	0.267	0.090	-0.182	0.009	0.319	-0.543	-0.359	0.121	0.196	-0.434	0.105	0.127

p_acetic	benzoic	c_coumaric	benzaldehyde	c_ferulic	vanillic	trux_FA	trux_CA	totp_unknown	totsugars	totphenolics	proto_aldehyde	corr_lignin	ash	d804	d88	benzofuran	d85	d55	node	leaf	inter	ear
-0.270																						
0.374	0.358																					
-0.195	0.752	0.336																				
-0.632	0.363	-0.320	0.361																			
-0.691	0.298	-0.363	0.315	0.430																		
-0.603	-0.236	-0.627	-0.312	0.205	0.729																	
0.644	0.286	0.802	0.328	-0.311	-0.530	-0.778																
-0.065	0.717	0.484	0.852	0.261	0.312	-0.298	0.454															
0.623	-0.278	0.101	-0.209	-0.409	-0.401	-0.194	0.416	-0.201														
-0.085	0.741	0.474	0.863	0.273	0.326	-0.291	0.441	0.999	-0.219													
-0.709	-0.048	-0.540	-0.083	0.371	0.736	0.832	-0.677	-0.186	-0.393	-0.167												
0.413	-0.131	0.105	0.028	-0.214	-0.550	-0.413	0.451	-0.208	0.654	-0.209	-0.351											
0.345	0.245	0.449	0.102	-0.211	-0.351	-0.387	0.332	-0.013	0.090	-0.008	-0.284	0.057										
-0.443	0.297	-0.050	0.334	0.356	0.768	0.487	-0.271	0.535	-0.437	0.537	0.475	-0.766	-0.173									
-0.081	0.383	0.154	0.223	0.217	0.507	0.279	0.104	0.415	0.249	0.412	0.138	-0.213	-0.105	0.528								
-0.438	0.184	-0.273	0.093	0.189	0.850	0.700	-0.386	0.285	-0.204	0.293	0.623	-0.616	-0.363	0.812	0.623							
-0.388	0.325	-0.071	0.311	0.367	0.757	0.525	-0.099	0.376	-0.016	0.387	0.573	-0.231	-0.298	0.677	0.755	0.766						
-0.207	0.593	0.321	0.558	0.246	0.306	-0.058	0.099	0.596	-0.472	0.611	0.102	-0.466	0.396	0.637	0.163	0.262	0.276					
-0.674	0.062	-0.544	0.106	0.349	0.602	0.546	-0.463	-0.108	-0.176	-0.092	0.654	-0.050	-0.467	0.160	0.005	0.400	0.404	-0.167				
-0.368	-0.069	-0.048	0.060	0.430	0.193	0.179	-0.306	0.084	-0.675	0.080	0.338	-0.532	0.098	0.484	-0.200	0.033	0.039	0.449	-0.097			
0.368	-0.102	-0.088	-0.188	-0.386	-0.284	-0.072	0.067	-0.251	0.604	-0.254	-0.267	0.518	0.052	-0.542	0.055	-0.257	-0.244	-0.435	-0.135	-0.701		
0.091	0.168	0.140	0.108	-0.174	0.057	-0.132	0.329	0.149	0.270	0.155	-0.156	0.167	-0.212	-0.053	0.206	0.245	0.224	-0.150	0.297	-0.592	-0.155	
